# Dynamically tunable band stop filter enabled by the metal-graphene metamaterials

**DOI:** 10.1038/s41598-018-21085-7

**Published:** 2018-02-12

**Authors:** Yan Liu, Renbin Zhong, Zhen Lian, Chen Bu, Shenggang Liu

**Affiliations:** 1 Terahertz Research Centre, School of Physical Electronics, University of Electronic Science and Technology of China, Chengdu, Sichuan 610054 China; 2 Cooperative Innovation Centre of Terahertz Science, Chengdu, Sichuan 610054 China; 3 State Key Laboratory of Millimeter Waves, City University of Hong Kong, 83 Tat Chee Av., Kowloon, SAR, Hong Kong, P.R. China

## Abstract

Dynamically tunable band stop filter based on metal-graphene metamaterials is proposed and numerically investigated at mid-infrared frequencies. The proposed filter is constructed by unit cells with simple gold strips on the stack of monolayer graphene and the substrate of BaF_2_. A stable modulation depth up to −23.26 dB can be achieved. Due to the cooperative effect of the “bright-bright” elements, the amount of the gold strips in each unit cell determines the number of the stop-bands, providing a simple and flexible approach to develop multispectral devices. Further investigations illustrate that the location of the stop bands not only can be adjusted by varying the length of gold strips, but also can be dynamically controlled by tuning the Fermi energy level of graphene, and deep modulation is acquired through designing the carrier mobility. With the sensitivity as high as 2393 nm/RIU of the resonances to the varieties of surrounding medium, the structure is also enabled to be an index based sensor. The results will benefit the on plane or integrated micro-structure research with simple structure and flexible tunability, and can be applied in multi-band stop filters, sensors and other graphene-based multispectral devices.

## Introduction

Electromagnetic (EM) metamaterials, a kind of artificially structured EM materials, are usually composed of periodically arranged subwavelength microstructures or ‘meta-molecules’, and have attracted great attention for their unique EM properties that the natural materials cannot realize. Some of the most typical meta-molecules are the metal split ring resonators^1–3^, the metal cut wires^4–6^ and the concentric rings^7–9^. By controlling the geometry of microelements in the metamaterial structures, the electric permittivity and magnetic permeability can be arbitrarily designed. Recently, the combination of metamaterials and plasmonics reveals an analogous physical phenomena of EM induced transparency (EIT), which is termed as plasmon induced transparency^5^ (PIT). The unit cell of PIT based metamaterials usually consists of bright mode and dark mode resonators^10^. The bright mode or super-radiant mode can be excited directly by an external incident field, and has a large scattering cross section^5^. In contrast, the dark mode or sub-radiant mode cannot be directly excited, but it can be excited by the local field of the bright mode via near field coupling and exhibits a larger quality factor due to the weak radiation coupling^11^.

Metamaterials with multiple PIT windows have many promising applications in optical information processing systems, effective switchers and ultrasensitive sensors. Traditional PIT based multispectral devices are usually designed by two approaches: the bright to bright mode coupling^12–14^ and the bright to dark mode coupling^5,15,16^, but usually come with an unsatisfactory modulation depth. Moreover, the optical properties of metamaterials based on metallic structures cannot be changed once the structure is fabricated. To improve above situation, some actively controlled elements, such as liquid crystals^17–19^, liquid metals^20,21^ and semiconductors^14^, are applied in these metamaterials devices. In recent years, graphene, a flat monolayer of carbon atoms packed into a dense 2D honeycomb crystal lattice, has become a very promising material for its unique electrical properties^22–24^, such as high electron mobility, flexible tunability, relatively low loss and tight field confinement. The most attractive one among these properties is that the conductivity of graphene can be dynamically tuned by changing the Fermi energy through chemical doping^25,26^ or electrostatic gating^27^. Therefore, the combination of graphene and metamaterials can provide an effective approach to improve the dynamically tunable resonant properties of the multispectral devices^28–31^.

In this work, by introducing a monolayer of graphene into a simple metamaterial structure, dynamically tunable dual-band and multi-band stop filters are proposed and researched. With two gold strips acting as typical bright mode resonators, a dual-band stop filter with an ideal modulation depth as high as −23.26 dB is achieved. To reveal the cooperative effect of the bright mode resonators, the electric field and surface current distributions are further investigated. The number of the stop-band can be easily increased by adding the gold strips in each unit cell, hence a filter with multi-band stop is obtained. The dynamically tunable properties of the proposed filters are realized by adjusting the graphene Fermi energy, and the modulation depth can be designed through varying the carrier mobility. Furthermore, the proposed structure can also be used as an index based sensor for its ultra-high sensitivity to the surrounding medium. All these results demonstrated that the presented metal-graphene based metamaterials provide promising applications in multi-band stop filters, sensors and other graphene-based multispectral devices.

## Results and Discussion

### Model construction of dual-band stop filter

**Figure 1:**
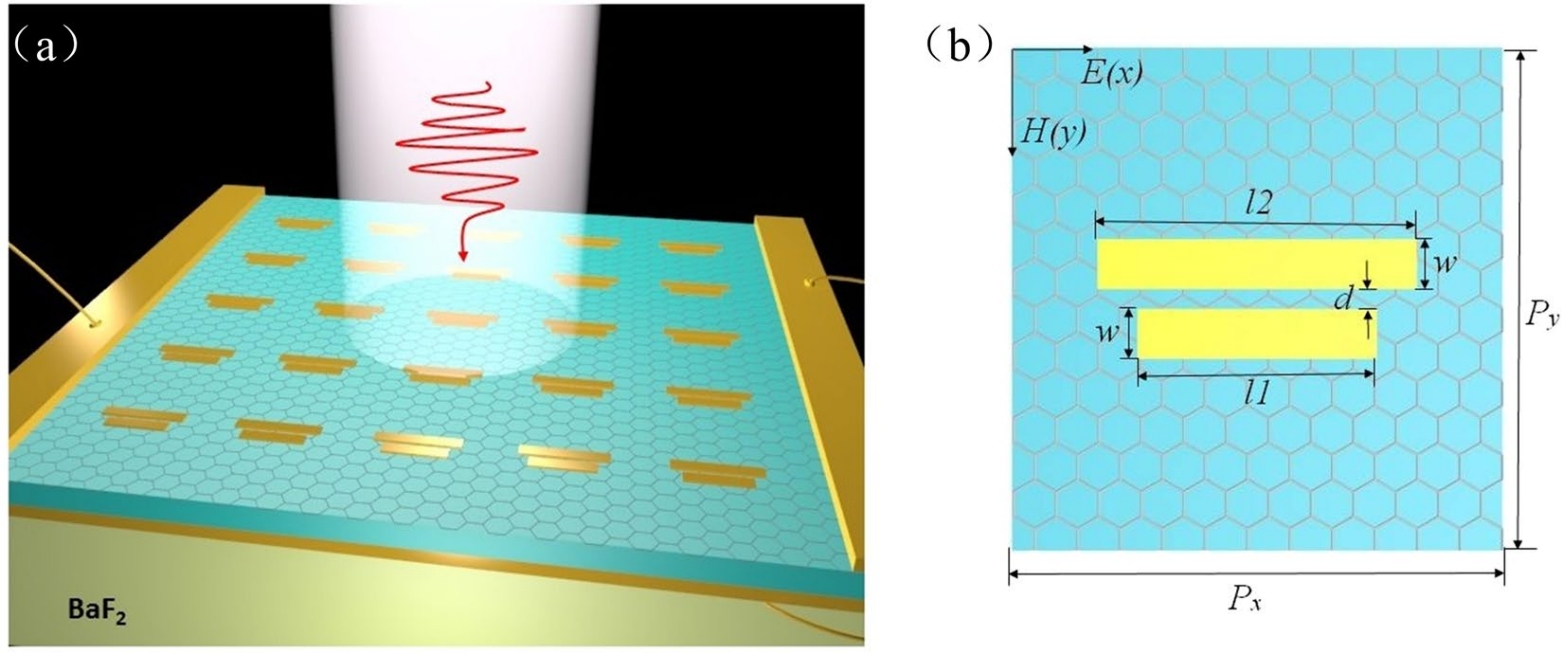
(**a**) Schematic of the proposed dual-band stop filter based on metal-graphene metamaterials and the incident light polarization configuration. (**b**) Top view of the unit cell structure. The geometrical parameters are as follows: *l1* = 2.0 μm, *l2* = 2.8 μm, *w* = 0.5 μm, *d* = 0.2 um, *P*_*x*_ = *P*_*y*_ = 4.2 μm, respectively.

The proposed dual-band stop filter is schematically depicted in Fig. 1(a). The periodically arranged gold strips and the BaF_2_ substrate assuming the refractive index *n* = 1.46 are separated by a monolayer of graphene and a thin layer of SU-8 photoresist. As shown in Fig. 1(b), two parallel gold strips with an identical width of *w* = 0.5 μm are symmetrically attached on the center of the unit cell and separated from each other by a small gap *d* = 0.2 μm. The lengths of the short and long gold strips are set as *l1* and *l2*, respectively. The periods of the unit cell are 4.2 μm both on x and y directions. The top metallic pad serves as the electrodes along with the square metallic ring at the back of the dielectric layer to tune the Fermi energy level of the graphene by applying a gate voltage. The thickness of the gold strips and the SU-8 photoresist layer are 75 nm and 300 nm, respectively.

In the mid-infrared region, the complex conductivity of monolayer graphene, consisting of interband and intraband contributions, can be given by Kubo formula^32,33^:

1 $${\sigma }_{g}(\omega ,\tau ,{\mu }_{c})={\sigma }_{\mathrm{int}ra}(\omega ,\tau ,{\mu }_{c})+{\sigma }_{\mathrm{int}er}(\omega ,\tau ,{\mu }_{c}).$$

with

2 $${\sigma }_{{\rm{inter}}}(\omega ,\tau ,{\mu }_{c})\approx \frac{j{e}^{2}}{4\pi \hslash }\,\mathrm{ln}[\frac{2|{\mu }_{c}|-(w+j/\tau )\hslash }{2|{\mu }_{c}|+(w+j/\tau )\hslash }],$$

3 $${\sigma }_{{\rm{intra}}}(\omega ,\tau ,{\mu }_{c})\approx j\frac{{e}^{2}{k}_{B}T}{\pi {\hslash }^{2}(w+j{\tau }^{-1})}[\frac{{\mu }_{c}}{{k}_{B}T}+2\,\mathrm{ln}(\exp (-\frac{{\mu }_{c}}{{k}_{B}T})+1)].$$

where *w*, *k*_*B*_, $\hslash$ and *e* is the incident angular frequency, Boltzmann constant, reduced Planck constant and electron charge, respectively. *T* is the temperature in kelvin, which is fixed to 300 K in this study. The value of chemical potential *μ*_*c*_ is equal to that of the Fermi energy level *E*_*f*_ when *k*_*B*_ < *μ*_*c*_, which can be written as ${E}_{f}=\hslash {\upsilon }_{F}\sqrt{\pi n}$, where *n* is the carrier concentration and the Fermi velocity is ${\upsilon }_{F}$ = 10^6^ m/s. Hence the Fermi energy is determined by the carrier concentration, which is proportional to the gate voltage *V*_*g*_ on the graphene as *n* = *ε*_*d*_*ε*_0_*V*_*g*_*/eh*, where *ε*_*d*_ and *h* is the permittivity and thickness of the insulated substrate material. The relaxation time $\tau =\mu {E}_{f}{e}^{-1}{{\upsilon }_{F}}^{-2}$ characterizes the plasmon decay on account of impurities, where *μ* is the carrier mobility. Recent literatures showed that the graphene carrier mobility could reach higher than 10^5^ cm^2^V^−1^s^−1^ in the experiment^34^, and is set as 2050 cm^2^V^−1^s^−1^ corresponding to the relaxation time $\tau =0.0205\,ps$ for *E*_*f*_ = 0.1 eV.

### The resonance mechanism

**Figure 2:**
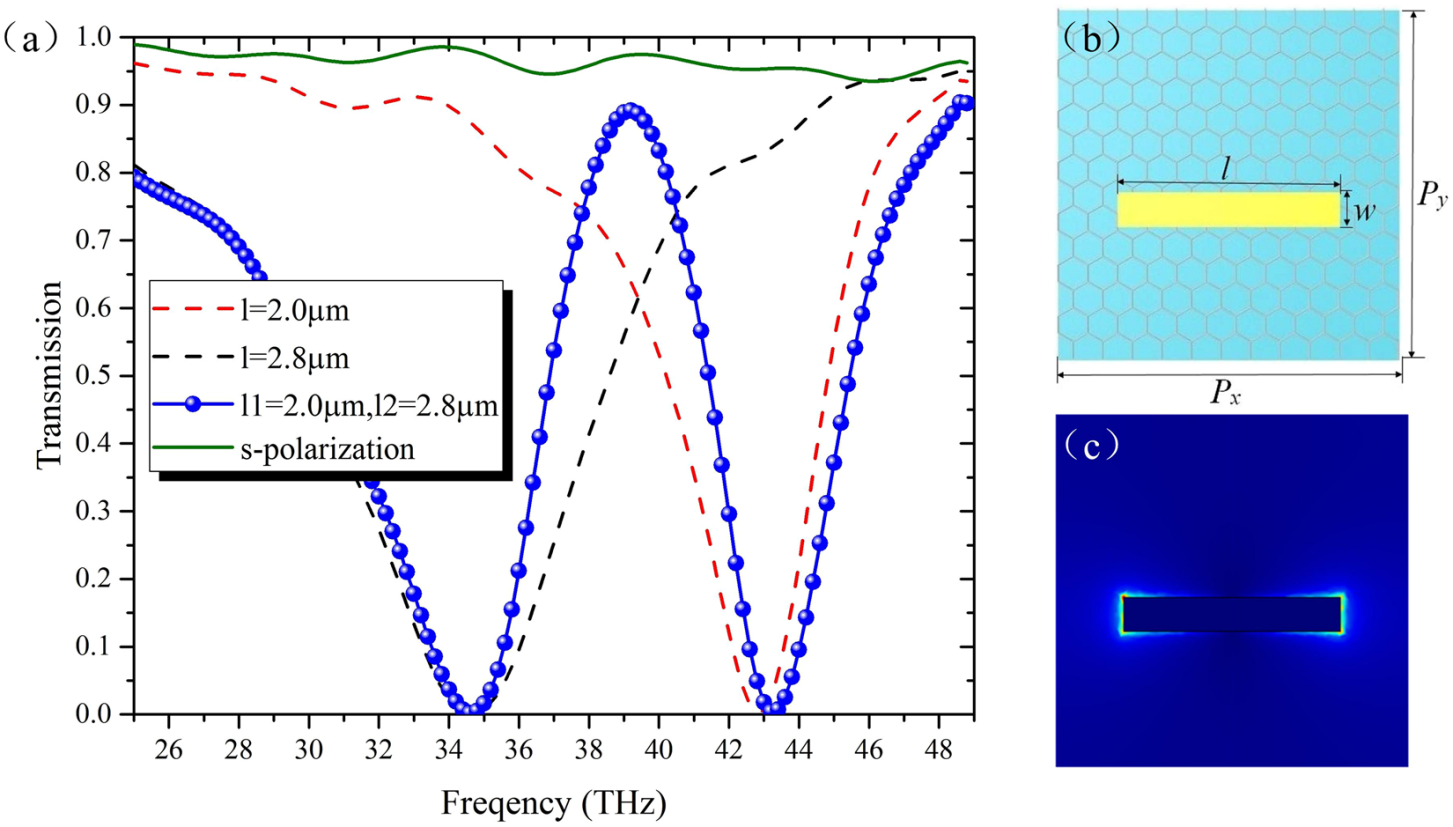
(**a**) The simulated transmission spectrum of metamaterials composed of the only single gold strip structure with different gold strip length *l* (dashed lines), and the double gold strips structure (blue balls) excited by a p-polarized incident light. The solid line illustrate the transmission spectrum of the proposed metamaterials excited by an s-polarized incident light. (**b**) The Schematic of the unit cell with only single gold strip structure. (**c**) The amplitude of electric field |E| for the only single gold strip structure. The Fermi energy of graphene is fixed as 0.1 eV.

A plane wave polarized parallel to the *x*-direction (p-polarized) is used as the normal incident light. Figure 2(a) shows the calculated transmission spectrum of the metal-graphene based filter with dual-band stop (blue balls), and two deep transmission dips are observed at 34.6 THz and 43.3 THz. The modulation depth, which is defined as the difference of the dB value between the transmission dip and peak, is as high as −23.26 dB. As contrast, the transmission spectrum of the proposed structure with s-polarized normal incidence is also depicted by the green solid curve. Obviously, it is not excited under this excitation. To reveal the resonance mechanism of the proposed structure, further investigations are conducted for the metamaterials composed of only single gold strip (see Fig. 2(b)) with the same parameters as in Fig. 1(b). The distribution of electric field amplitude |E| is shown in Fig. 2(c) at its resonance frequency. One can see that the gold strip is strongly excited and the electric fields concentrate mostly around its edges and ends, which is a typical electric dipolar mode distribution. The metal strips with an electric dipolar mode pattern, as we know, are typical ‘bright’ elements for they can be excited directly by the incident field, with a large scattering cross section and a low quality factor^5^. As depicted by the dash curves in Fig. 2(a), the only single gold strip structures with *l* = 2.8 μm (black) and 2.0 μm (red) show strong resonance dips at 34.6 THz and 43.3 THz, respectively, which are exactly corresponding to the two resonances of the proposed filter. So, different from the traditional PIT effect of near-field coupling between bright elements and black elements, both the short and long gold strips in the dual-band stop filter are act as bright elements, and the two deep transmission dips are actually resulted from the simple cooperative effect of the two bright elements.

**Figure 3:**
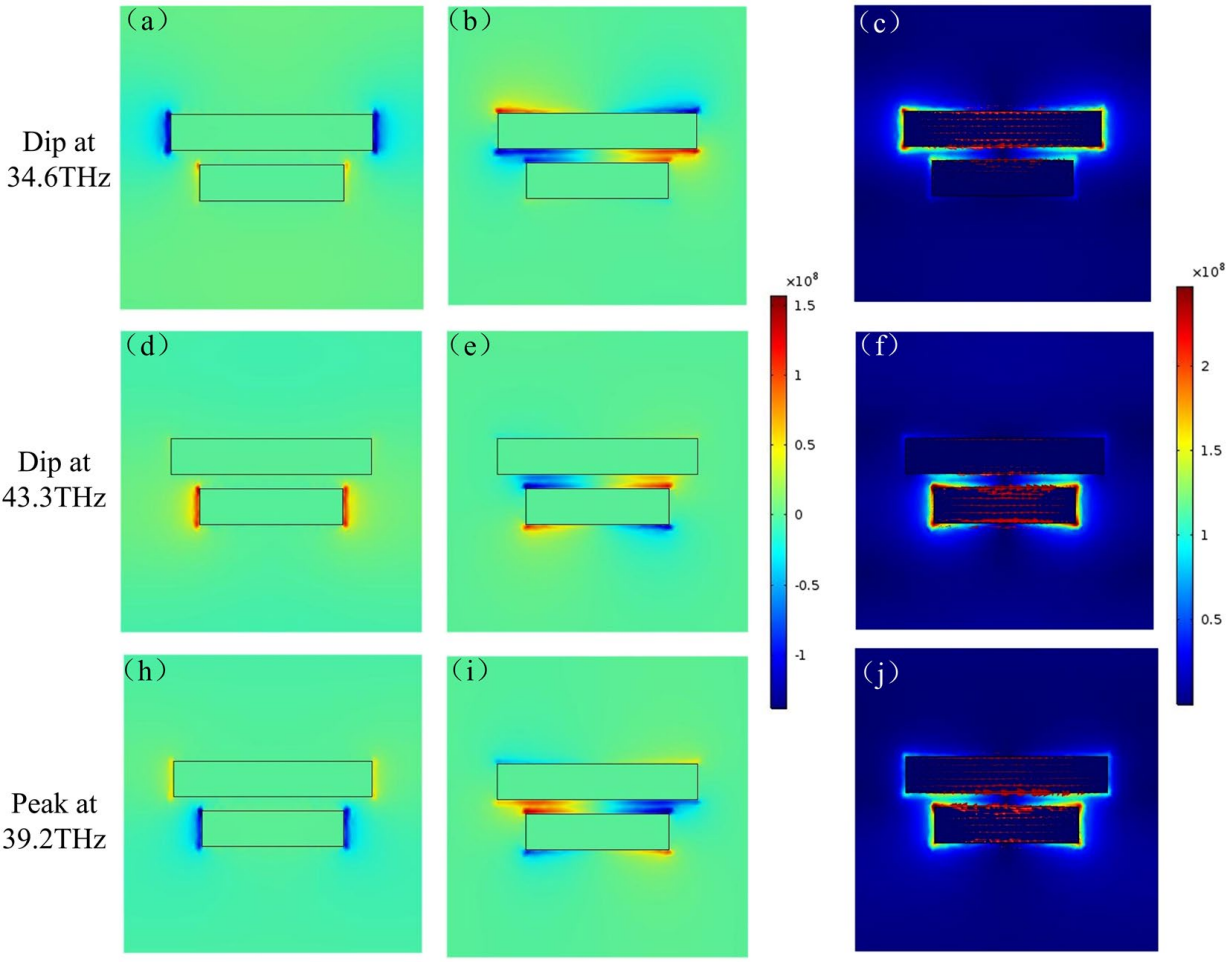
Distributions of the x-component electric field ***E***_*x*_ (first column), the y-component electric field ***E***_*y*_ (second column), and the amplitude of electric field |***E***| with current density (third column), at resonance frequencies of 34.6 THz (first row), 39.2 THz (second row) and non-resonance frequency 43.3 THz (third row), respectively.

In order to further demonstrate the cooperative effect of the gold strips in the proposed structure, distributions of *x*-component electric field *E*_*x*_, *y*-component electric field *E*_*y*_ and the amplitude of electric field |E| with surface current density are calculated and depicted in Fig. 3 at three different frequencies, i.e., 34.6 THz, 43.3 THz and 39.2 THz, as typical frequencies presenting resonances and non-resonance of the proposed dual-band stop filter. At the low-order transmission dip of 34.6 THz (Fig. 3(a)–(c)), both the long and short gold strips are excited, while the electric field concentrates almost entirely at the edges and ends of the long gold strip. Figure 3(c) shows that there are high density of surface current (red rows) travelling along the metal strip. According to Faraday law of electromagnetic induction, the induced magnetic flux from the long gold strip excites induced currents on the short gold strip with opposite flowing direction of the directly exciting current, meanwhile weakens the surface current on the short gold strip. As a result, the proposed structure shows the resonance characteristics in accordance with the long gold strip. For the electric field distribution at the high-order transmission dip of 43.3 THz (Fig. 3(d)–(f)), it is evident that the short gold strip is strongly excited, while the excitation of the long gold strip is relatively weak, hence the unit cell mainly exhibits the resonance characteristics of the short gold strip. As for the transmission peak at 39.2 THz (Fig. 3(h)–(j)), the electric field is much weaker compared to the distributions at other frequencies, and there is no obvious near-field coupling between the short and long gold strips in the dual-band stop filter. The surface current along the long gold strip have an opposite direction of the short one, and destructively combine with the induced current densities from each other, leading to a broad transmission band.

**Figure 4:**
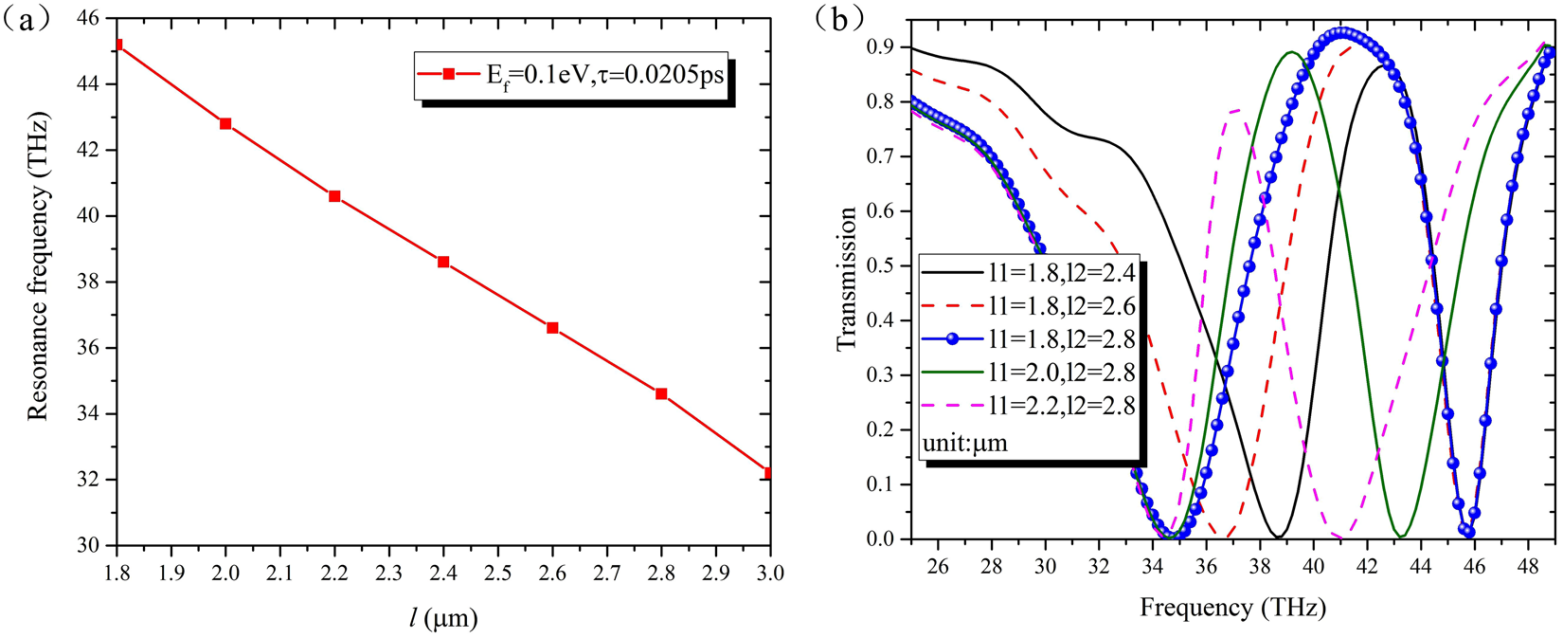
(**a**) The shift of resonance frequency as a function of strip length *l* for the only single gold strip structure. (**b**) The simulated transmission spectrum for various strips lengths. All the results are calculated with *E*_*f*_ = 0.1 eV, *τ* = 0.0205 ps.

As we noted above, the length of gold strip plays a key role in the location of the transmission dip. To explore this relationship, the resonance frequencies of the single gold strip structure versus its length are displayed in Fig. 4(a). It is evident that the increment of strip’s length *l* leads to a significant red shift of the resonance frequency, suggesting that the required stop band can be reached by designing the special length of the gold strips. Figure 4(b) shows the transmission spectrums of the proposed filter with various location of the stop bands by changing the length of gold strips. It can be seen that a relatively stable modulation depth around −23.26 dB can be obtained despite the variation of the gold strip’s length. The center frequency of the low-order stop band is red shifted from 38.6 THz to 34.6 THz as the long gold strip’s length *l2* increases from 2.4 μm to 2.8 μm, while the higher one remains unchanged for the fixed length of short gold strip. Similarly, the varieties of the short gold strip length *l1* decreasing from 2.2 μm to 1.8 μm corresponds to a blue shift of the high-order stop band, while the location of the low-order stop band keeps as a constant because of the fixed length *l2*. The shift of the resonance frequency results a broader transmission band. All of the results are in good agreement with those in Fig. 4(a).

### The dynamically tunable properties enabled by the monolayer graphene

**Figure 5:**
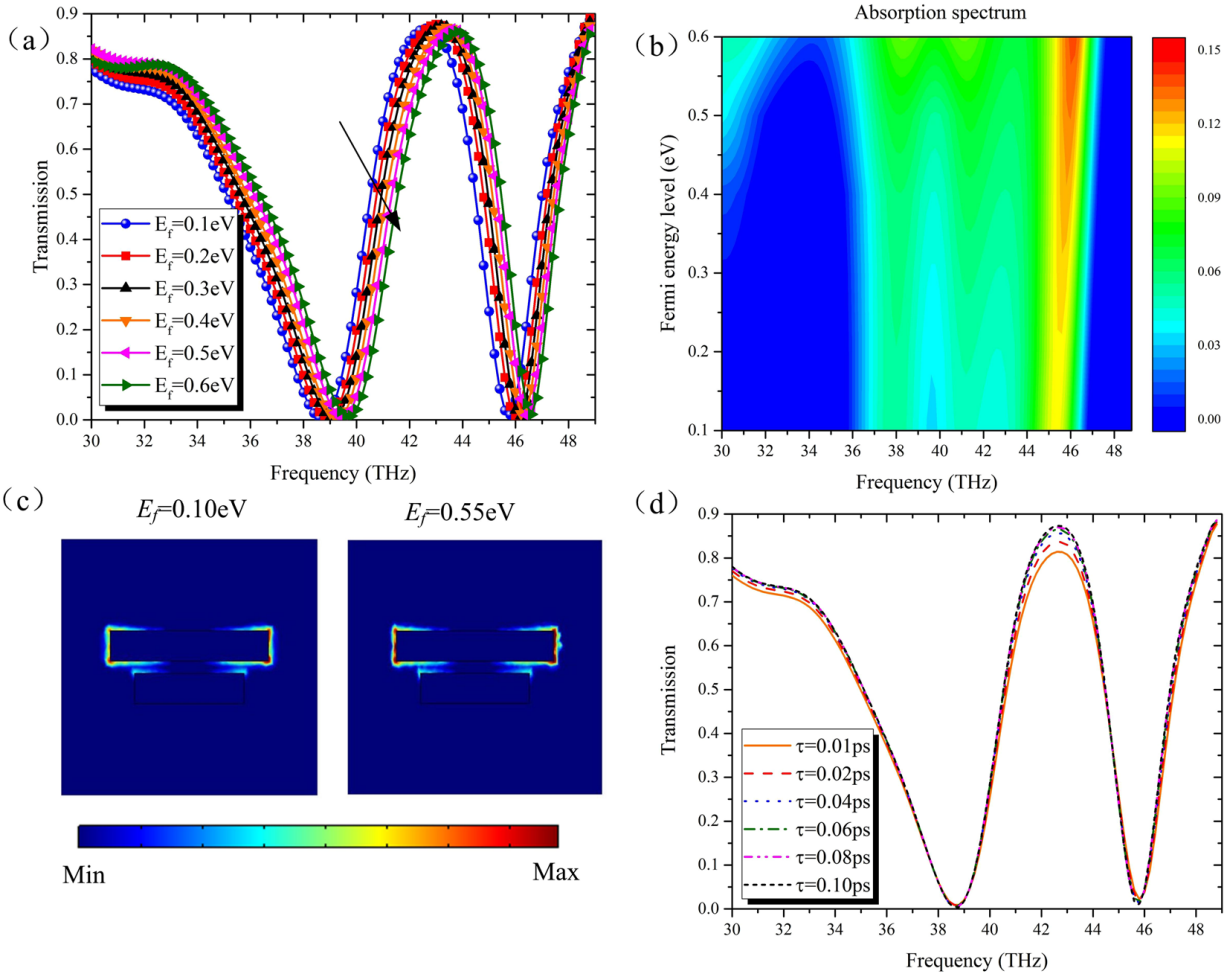
For the dual-band stop filter, the calculated (**a**) transmission spectrum and (**b**) absorption map with different Fermi energies and fixed relaxation time *τ* = 0.0205 ps. The black arrow indicates the growing trend for Fermi energy. (**c**) The distributions of electric field |E| at the center frequencies of the low-order stop bands with *E*_*f*_ = 0.1 eV (left) and 0.55 eV (right). (**d**) Transmission spectrum for different relaxation times *τ* from 0.01 ps to 0.1 ps. The Fermi energy of graphene is fixed as 0.1 eV. The red arrow indicates the growing trend versus the relaxation time. The length of the two gold strips are 1.8 μm and 2.4 μm.

As discussed above, the graphene conductivity is a function of the Fermi energy, which can be flexibly tuned by the applied gate voltage between two metallic pads. Figure 5(a) and (b) illustrate the simulated transmission and absorption spectrums of the presented dual-band stop filter when the Fermi energy changes from 0.1 eV to 0.6 eV. For a normal incidence, the absorption coefficient is given by *A*(*f*) = 1 − *R*(*f*) − *T*(*f*), where *T*(*f*) and *R*(*f*) is the transmission and reflection coefficient. As shown in Fig. 5(a), the transmission peak exhibits a clear blue shift and the modulation depth shows a slight reduction as the Fermi energy increases. Meanwhile, it is obviously that the amplitude of absorption increases as the Fermi energy increases (see Fig. 5(b)). This variety can be explained as follows: The high Fermi energy results from a large carrier concentration, which contributions to the enhancement of plasmons oscillation, leading to a stronger field confinement. As a result, the absorption coefficient increases. To corroborate this association, Fig. 5(c) depicts the electric field distributions with *E*_*f*_ = 0.1 eV and 0.55 eV at the corresponding center frequencies of the low-order stop bands. As expected, stronger confined electric fields are observed around the long gold strip at the Fermi energy of 0.55 eV.

Though the structure couldn’t be dynamically adjusted by graphene carrier mobility in the experiment, the modulation depth can be further improved by choosing the monolayer graphene with a high value. When the Fermi energy of graphene is fixed as 0.1 eV, Fig. 5(d) shows the transmission spectrums of the proposed filter with various relaxation times *τ* ranging from 0.01 ps to 0.1 ps, corresponding to the values of the graphene carrier mobility *μ* changing from 1000 cm^2^V^−1^s^−1^ to 10000 cm^2^V^−1^s^−1^. It is evident that the increasing of graphene carrier mobility has no effect on the location of the resonance frequencies, but raises the peak of the transmission band contributing to the increment of the modulation depth, due to the reduction of the graphene absorption. Thus, by introducing the monolayer graphene into the filter, its stop bands can realize dynamical tunability through controlling the graphene Fermi energy, and then a relatively good modulation depth can be achieved by designing the carrier mobility of graphene.

### Further application as an index based sensor and extended structure of the multispectral devices

**Figure 6:**
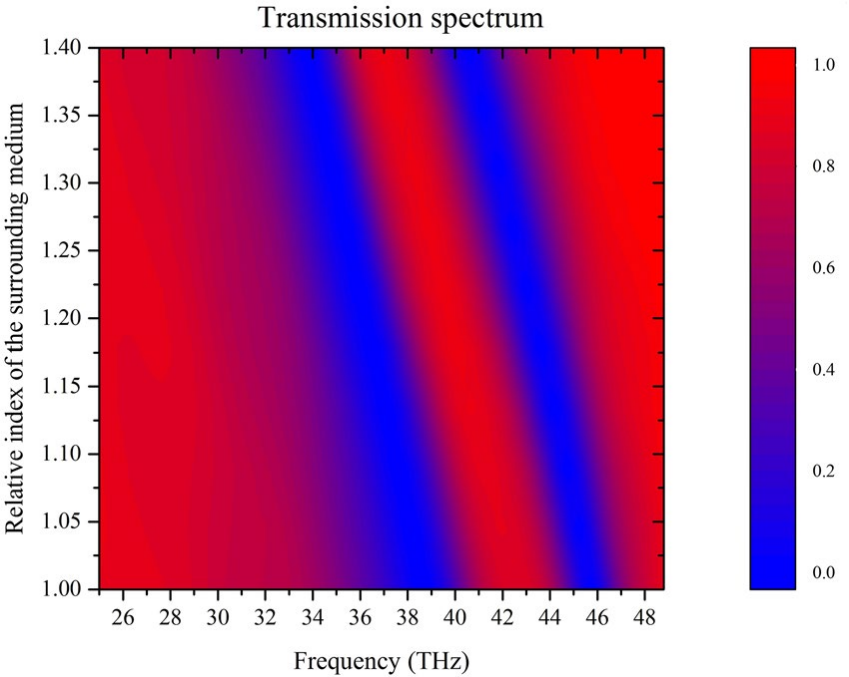
Calculated transmission map for different refractive index of the surrounding medium ranging from 1.0 to 1.4. The relaxation time and Fermi energy of graphene are set as *τ* = 0.0205 ps and *E*_*f*_ = 0.1 eV.

Note that the resonance frequencies of the proposed structure are sensitive to the refractive index of the surrounding medium. Figure 6 shows the transmission spectrum of the proposed structure with the refractive index of the surrounding medium varying from 1.0 to 1.4 continuously. There is a conspicuous red shift of the resonance frequencies with the increasing of the refractive index. The sensitivity of the sensors is defined as the position of the transmission peak shift over the refractive index change unit (RIU), here. For example, when the change unit is set as 0.1, the minimal shift of the wavelength for the transmission peak is 239.3 nm, hence the sensitivity of the proposed structure is as high as 2393 nm/RIU or more. Therefore, one can exactly estimate the surrounding medium by the variety of the transmission spectrum, and it can be used for the design of refractive index based sensors.

**Figure 7:**
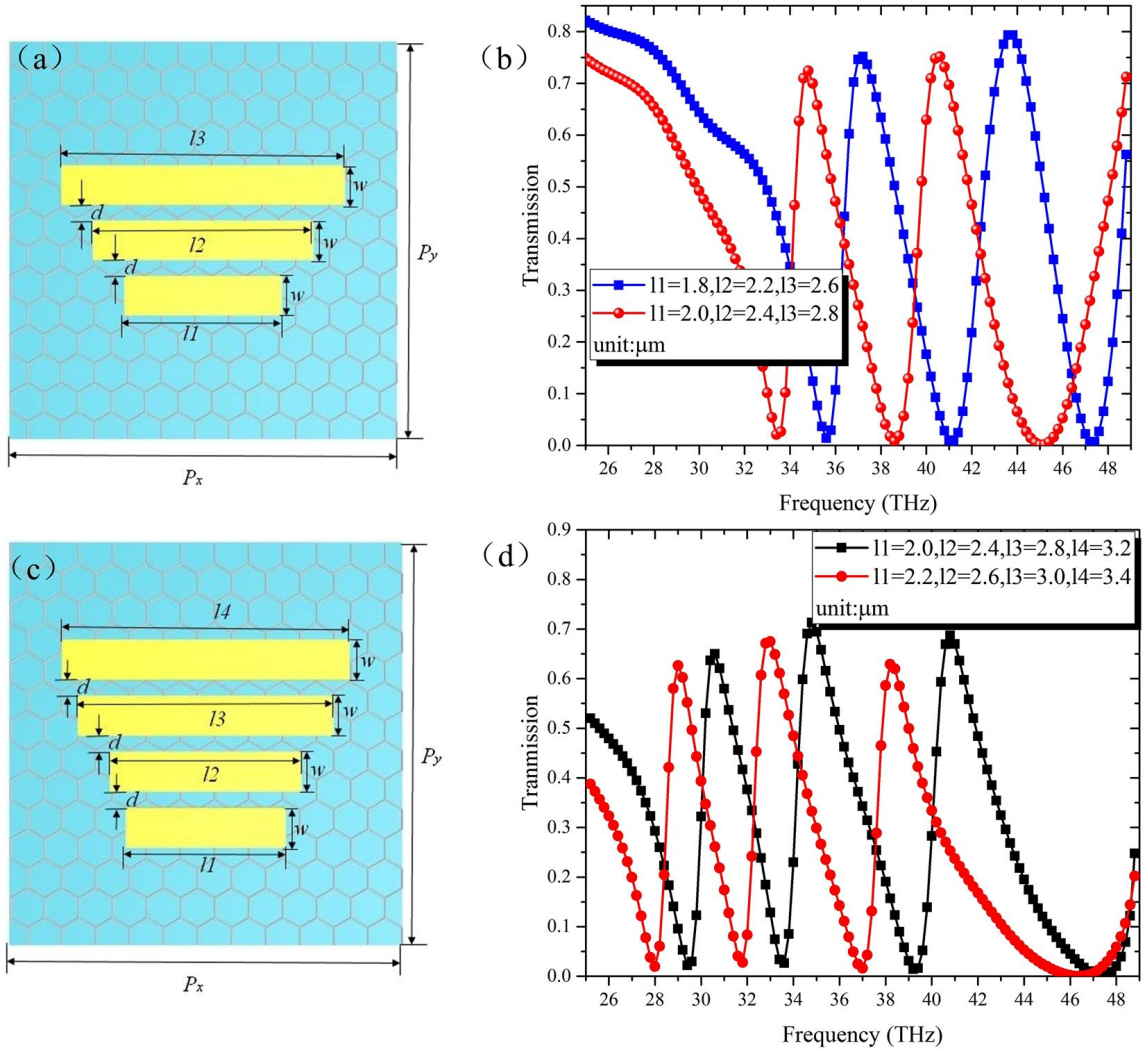
For the three-band stop filter: (**a**) Unit cell and (**b**) the simulated transmission spectrum; For the four-band stop filter: (**c**) unit cell and (**d**) the simulated transmission spectrum. The Fermi energy of graphene is fixed as 0.1 eV, other dimensions are as follows: *w* = 0.5 μm, *d* = 0.2 μm, *P*_*x*_ = *P*_*y*_ = 4.2 μm, respectively.

Finally, multispectral metamaterials are realized with the Fermi energy and relaxation time are fixed as 0.1 eV and 0.0629 ps, respectively. The diagram of the structure with triple-band stop are shown in Fig. 7(a), and the geometric parameters are consistent with those in Fig. 2(b) except the lengths of gold strips. As shown in Fig. 7(b), three transmission dips emerge in the spectrum at 47.2 THz, 41.2 THz and 35.6 THz, when gold strips’ lengths are *l1* = 1.8 μm, *l2* = 2.2 μm and *l3* = 2.6 μm. Similarly, the four-band stop structure can be obtained simply by increase the number of gold strips to four as depicted in Fig. 7(c), and the corresponding transmission spectrum is shown in Fig. 7(d). Likewise, more multispectral metamaterials can be realized by adding gold strips with requisite lengths. Hence, the proposed structure can be designed and fabricated more easily compared with other metamaterials composed of complex unit cells, especially for the realization of multispectral devices.

## Conclusions

In conclusion, dynamically tunable band stop filters based on metal-graphene metamaterials are investigated at mid-infrared frequencies. The calculated transmission spectrums exhibit broad stop bands, and the number of the stop-band can be increased simply by adding the gold strips in each unit cell. The center frequency of the stop band or the location of resonance not only can be adjusted by varying the length of gold strips, but also can be dynamically controlled through the graphene Fermi energy. The increment of graphene carrier mobility will enhance the amplitude of the transmission peak to realize low loss transmission in pass band. Furthermore, the proposed structure can also be used as an index based sensor for its high sensitivity to the surrounding medium. The results will benefit the on plane or integrated micro-structure research with simple structure and flexible tunability, can be applied in multi-band stop filters, sensors and other graphene-based multispectral devices.

## Methods

The proposed structures are numerically calculated using finite element method (FEM). In the three-dimensional simulations, Floquet ports with perfectly matched layer (PML) absorbing boundary conditions are used in the z-direction, and the unit cell both in *x* and *y* directions is performed with the periodic boundary condition. A p-polarized plane wave is used as the incident light. The conductivity of the gold is described by the Drude model with the plasma frequency *w*_*p*_ = 1.36 × 10^16^ rad/s and the scattering rate *Γ* = 3.33 × 10^13^ rad/s in this work. The monolayer graphene is modeled as a surface current in the boundary conditions, which is defined as the product of graphene conductivity and the electric field in the frequency domain. The surrounding medium are assume to be uniform with the refractive index *n*_*r*_ = 1. The transmission coefficient *T*(*f*) and reflection coefficient *R*(*f*) are calculated from S-parameters in the simulated results, and given as *T*(*f*) = |S_21_|^2^, *R*(*f*) = |S_11_|^2^ respectively.
